# Books Reviewed

**Published:** 1866-02

**Authors:** 


### Books Reviewed.

The Practice of Medicine. By Thomas Hawks Tanner, M. D., F. L. S. From the fifth London edition. Enlarged and improved. Philadelphia: Lindsay & Blakiston. 1866.

We are pleased to see that this highly practical work is presented to the profession of this country; an author who treats disease in such a manner as to be followed as a guide with any degree of safety should be heartily thanked, and applauded even, by the profession generally. We have books upon the practice of medicine in great abundance, and most of them are valuable on many accounts; but to say that any one has, as yet, indicated a proper plan of treating disease, even though he may have faithfully described it, is more than the truth will warrant. Who follows Wood or Watson in the treatment of disease? No one of any considerable experience. This work covers a wide range of subjects, all treated in a brief, concise, practical manner. The author shows familiarity with all that has been learned both of the nature and treatment of disease, but has not attempted to reproduce it, or a synopsis of it even, but has furnished a complete and comprehensive work upon the practice of medicine. We should be pleased to quote largely from our author upon many practical points, but our space will not admit of it at present, and we willingly omit it in expectation that all our readers will read the book for themselves. The subject of Cholera and its transmissibility being introduced so frequently in our pages, induces us to make one quotation upon that subject:

"The only explanation which can be given of the cause of cholera is, that it is due to some *materies morbi*—a septic agent—the existence, increase, power and transmission of which from place to place is favored by some particular state of the atmosphere, associated, probably, with a high temperature. The action of the poison is undoubtedly encouraged by filth of all kinds. As far as I can glean from the recorded evidence—and I have carefully studied the subject—it certainly appears to me to be, to a certain degree, contagious; in other words, I believe that human intercourse has a share in propagating the disease, though it is not the only means of effecting its diffusion. We must remember, however, that cholera, like other contagious disorders, can only be taken by a person predisposed to disease; we may indeed compare a contagious or infectious disorder to a seed, which, unless put into a fit soil, undergoes no change—does not grow or take root."

---

Principles of Surgery. By James Syme, F. R. S. E.

We see by the Editor's Preface that an older pupil of Prof. Syme's than himself has said about as nice a thing of his teacher as could be said, and will preface what we have to say of the work by introducing the author in the words of Dr. John Brown, of Edinburgh, who says: "Everywhere personally, professionally, and publicly, reality is his aim and his attainment. He is one of the men—they are all too few—who desire to be on the side of truth rather than have truth on their side, and whose personal and private worth are always better understood than expressed. It has been happily said of him, that he never wastes a word, a drop of ink, or a drop of blood, and his is the strongest, exactest, truest, immediatist, safest intellect dedicated by its possessor to the surgical cure of mankind I have ever yet met with. He will, I believe, leave an inheritance of good done and mischief destroyed, of truth in theory and practice established, and of error in the same exposed and ended, such as no one, even John Hunter, has been gifted to bequeath to his fellow men.

"As an instrument for discovering truth I have never seen his perspicuity equalled. His mental eye is achromatic, and admits into the judging mind a pure, white light, and records an undisturbed, unclouded image, undiminished and unenlarged in its passage; and he has the moral power, courage and conscience to use and devote such an inestimable instrument aright."

Whoever has carefully read the Surgical Lectures of Prof. Syme for the past few years, is prepared to appreciate his Principles of Surgery, as well as the compliments of his admirers, and perhaps nothing more could be said, giving a clearer and more truthful idea both of the author and book, than has been already.

It appears that the Eclectic School of Medicine have published an American edition of Prof. Syme's Surgery, in mutilated form; its circulation has been limited to the adherents of this *ism*, and the profession have never been acquainted with its merits.

The present edition is in pure form, and the editor has conferred a great favor upon the profession of this country. The work comprises a general treatise upon surgery, with consideration of a wide range of topics. It contains near nine hundred pages; is in clear type, and published in substantial form. It will form a most admirable guide in practice, and be regarded as standard authority. The chapters upon Diseases of the Rectum, Excision of Diseased Joints, and Miscellaneous Clinical Observations, are especially worthy of mention, and greatly increase the value of a book otherwise devoted to consideration of the general principles and practice of surgery.

---

The Use of the Larynoscope in Diseases of the Throat. With an Appendix on Rhinoscopy. By Morell Mackenzie, M. D., Lond., M. R. C. P. Philadelphia: Lindsay & Blakiston. 1865.

So far as we are able to judge, this book will prove itself satisfactory authority upon the diseases it treats. The author makes no undue pretension, but gives a history of the art he describes, rendering honor to whom honor is due. The descriptions are plain and practical, and the illustrations numerous and well executed. The following is a list of the illustrations:

Bozzini's laryngeal speculum *(after Hufeland;)* Dr. Babington's laryngeal mirrors; Avery's laryngoscope; reflector attached to spectacle-frame; the light-concentrator; diagram showing the relative positions of the planes of the larynx and laryngeal mirror; the position of the hand and mirror, when the latter has been properly introduced for obtaining a view of the larynx; laryngoscopic drawing, showing the vocal cords drawn widely apart, and the position of the various parts above and below the glottis, during inspiration; laryngoscopic drawing, showing the approximation of the vocal cords, and the position of the various parts in the act of vocalization; Dr. Smyly's recipro-laryngoscope; the epiglottic pincette; the self-holder, or *fixateur;* the head-rest; jagged condition of the vocal cords, and a wart on the epiglottis; excrescences on the right ary-epiglottidean fold; Lewin's *pulverisateur;* the laryngeal galvanizer; the laryngeal lancet; chronic œdema of the larynx; the laryngeal forceps, scissors and *ecraseur;* excrescences in the larynx; excrescences on, and beneath, the vocal cords; a small wart on the right vocal cord; warts on the vocal cords; a small polypus, attached near the anterior insertion of the vocal cords; warts on the vocal cords.

The book contains, probably, about all that is really known upon the subject, and whoever desires to learn and practice in this department will not fail to carefully read this author.

---

On the Diseases, Injuries and Malformations of the Rectum and Anus; with Remarks on Habitual Constipation. By T. J. Ashton, with illustrations. Second American from the fourth and revised English edition. Philadelphia: Henry C. Lea, 1865.

This work is comprised in twenty chapters, upon the following subjects: irritation and itching of the anus; inflammation and excoriation of the anus; excressences of the anal region; contraction of the anus; fissure of the anus and lower part of the rectum; neuralgia of the anus and extremity of the rectum; inflammation of the rectum; ulceration of the rectum; hæmorrhoidal affections; enlargement of hæmorrhoidal veins; prolapsus of the rectum; abscess near the rectum; fistula in anus; polypi of the rectum; stricture of the rectum; malignant diseases of the rectum; injuries of the rectum; foreign bodies in the rectum; malformations of the rectum; habitual constipation.

We have given the subjects of the chapters in order to show that this work is a most practical one, and valuable to every practitioner. The last chapter upon habitual constipation is an exceedingly valuable one, and what he says upon this subject should be known by every physicion. The whole teaching is illustrated by cases which have occurred in the practice of the author or of other distinguished physicians, and in this way the value of the work is greatly augmented. We cannot say too much in favor of the book, which contains a most perfect digest of everything within the scope of its plan, and will prove a valuable addition to the library of every practitioner.

